# Avian infectious bronchitis virus disrupts the melanoma differentiation associated gene 5 (MDA5) signaling pathway by cleavage of the adaptor protein MAVS

**DOI:** 10.1186/s12917-017-1253-7

**Published:** 2017-11-13

**Authors:** Liping Yu, Xiaorong Zhang, Tianqi Wu, Jin Su, Yuyang Wang, Yuexin Wang, Baoyang Ruan, Xiaosai Niu, Yantao Wu

**Affiliations:** Jiangsu Co-Innovation Center for Prevention of Animal Infectious Diseases and Zoonoses, College of Veterinary Medicine, Yangzhou University, Yangzhou, Jiangsu 225009 China

**Keywords:** Infectious bronchitis virus, Melanoma differentiation associated gene 5, Retinoic acid-inducible gene-I, Mavs

## Abstract

**Background:**

Melanoma differentiation associated gene 5 (MDA5) and retinoic acid-inducible gene-I (RIG-I) selectively sense cytoplasmic viral RNA to induce an antiviral immune response. Infectious bronchitis virus (IBV) is one of the most important infectious agents in chickens, and in chicken cells, it can be recognized by MDA5 to activate interferon production. RIG-I is considered to be absent in chickens. However, the absence of RIG-I in chickens raises the question of whether this protein influences the antiviral immune response against IBV infection.

**Results:**

Here, we showed that chicken cells transfected with domestic goose RIG-I (dgRIG-I) exhibited increased IFN-β activity after IBV infection. We also found that IBV can cleave MAVS, an adaptor protein downstream of RIG-I and MDA5 that acts as a platform for antiviral innate immunity at an early stage of infection.

**Conclusions:**

Although chicken MDA5 (chMDA5) is functionally active during IBV infection, the absence of RIG-I may increase the susceptibility of chickens to IBV infection, and IBV may disrupt the activation of the host antiviral response through the cleavage of MAVS.

**Electronic supplementary material:**

The online version of this article (doi: 10.1186/s12917-017-1253-7) contains supplementary material, which is available to authorized users.

## Background

Infectious bronchitis (IB) is a serious and highly contagious disease in chickens that is caused by the infectious bronchitis virus (IBV) [1]. Although the host uses multiple mechanisms to thwart viral invasion, the overall clearance and outcome of IBV infection in chickens are critically dependent on the early protection provided by the innate immune system [2]. To enable its survival, IBV has evolved to disrupt the activation of the host antiviral signaling pathway using a number of mechanisms, such as delaying the activation of the IFN response during the early stages of IBV infection [3, 4].

The innate immune system plays a critical role in the detection and elimination of invading pathogens, especially the IFN antiviral immune response [5]. To activate the antiviral immune response, pattern recognition receptors (PRRs) recognize specific pathogen-associated molecular patterns (PAMPs) [6, 7]. The PRRs include Toll-like receptors (TLRs), retinoic acid-inducible gene I (RIG-I)-like receptors (RLRs), and nucleotide-binding oligomerization domain (NOD)-like receptors (NLRs). RLRs include RIG-I [8], melanoma differentiation associated gene 5 (MDA5) [9] and laboratory of genetics and physiology 2 (LGP2) [10]. RIG-I and MDA5 interact with the mitochondrial antiviral signaling gene (MAVS, also called IPS-1/VISA/CARDIF) [11], a critical downstream adaptor protein located at the mitochondrial membrane, via caspase activation and recruitment domains (CARD)-CARD domains at their N-terminal [12]. Activated MAVS can recruit downstream interferon regulatory factor-3/7 (IRF3/IRF7) and the transcriptional factor nuclear factor κB (NF-κB) [13], leading to the rapid production of type I IFNs and proinflammatory cytokines [11, 14, 15].

Although both RIG-I and MDA5 are closely related, exhibiting 25% and 40% identities in their N-terminal CARD and C-terminal helicase domains [16, 17], they can recognize different types of ligands and distinct subsets of RNA viruses. RIG-I has been reported to recognize short dsRNA produced during the replication of RNA viruses and uncapped 5′-triphosphate (5′-ppp) ssRNA [18]. MDA5 can be activated by long dsRNA, including the synthetic dsRNA analogue poly I:C [16, 19]. Overexpression of MDA5 and RIG-I inhibits the growth of encephalomyocarditis virus (EMCV) and vesicular stomatitis virus (VSV) [19], and overproduction of MDA5 but not RIG-I leads to enhanced IFN-β promoter activity in measles virus (MV)-infected A549 cells [9]. It has also been demonstrated that expression of MDA5 and RIG-I resulted in the activation of the IFN-β promoter in influenza A virus-infected epithelial cells [20]. Barber et al. suggested that the lack of RIG-I observed in chickens results in a deficiency of the antiviral innate immune response, possibly explaining the high susceptibility of chickens compared to ducks during Avian influenza virus (AIV) infection [21]. Similarly, the absence of RIG-I in chickens may contribute to the susceptibility of only chickens to IBV.

To explore the mechanisms that control the chicken immune response to IBV infection with regard to the RIG-like helicase, we have cloned chicken MDA5 (chMDA5) and domestic goose RIG-I (dgRIG-I) and demonstrated that they act as positive regulators in the activation of IFN-β induced by IBV. Furthermore, the knockdown or overexpression of chMDA5 has no effect on IBV replication. In this study, we also investigated the potential role of MAVS in the MDA5-mediated antiviral signaling pathway after IBV infection and demonstrated a positive regulatory role of MAVS.

## Methods

### Virus and cells

The JS/2010/12 strain of IBV was previously characterized as nephropathogenic by our laboratory, and its genomic sequence was determined (GenBank accession No. JQ900122.1). In this study, the stock of JS/2010/12 strain propagated in 10-day-old SPF Line 22 of White Leghorn chicken embryos for 5 passages (P5) was used. The 50% tissue culture infective dose (TCID50) of the IBV strain was determined by identifying the cytopathic effect (CPE) induced by the virus in CEK cells. The DF1 chicken fibroblast cell line was used for all transfection-based assays. The cells were maintained in Dulbecco’s modified Eagle’s medium (DMEM, HyClone) containing 10% FBS. CEK cells were aseptically generated from 20-day-old SPF chicken embryos. The cell suspension was obtained by trypsinization of kidneys for 30 min at 37 °C and subsequent filtration with a 100-μm mesh. Then, the cells were cultured in M199 media (HyClone) containing 3% FBS (HyClone).

### Plasmid and small interfering RNA (siRNA)

The chMDA5 ORF was amplified from CEK cells by overlap PCR with primers chMDA5-F1/R1 and chMDA5-F2/R2, producing a 3006 bp MDA5 PCR product (GenBank accession No. GU570144.1). The PCR product was digested with *Eco*R V and *Xba* I, then was inserted into the pcDNA-5′-Flag plasmid that had been digested with the same enzymes. The chTLR3 ORF was PCR amplified from CEK cells with the primer pair chTLR3 F/R, and the product was cloned into the p3 × flag-CMV-7.1 vector (Invitrogen) at the *Not* I and *Eco*R V sites. The dgRIG-I ORF (GenBank accession No. JF804977) was amplified from goose splenic cDNA using primers dgRIG-I F/R, and the fragment was inserted into the p3 × flag-CMV-7.1 vector at the *Eco*R I and *Bam*H I sites. The small interfering RNAs (siRNA) targeting the chicken chMDA5, chTLR3, and chMAVS mRNAs as well as control siRNA were synthesized by Santa Cruz Biotechnology and have been previously described [22].

### Transfection

Plasmids and siRNA were transfected into cells with Lipofectamine 2000 (Invitrogen) according to the manufacturer’s instructions. Briefly, plasmid DNA (2 μg for a 6-well plate) and siRNA (20 nM, Santa Cruz Biotechnology) were diluted with opti-MEM. Lipofectamine 2000 (5 μl for a 6-well plate) was also diluted with opti-MEM. Diluted DNA was added to the diluted Lipofectamine 2000 reagent (1:1) and was incubated for 5 min, then inoculated into cells and incubated for 24 h before further treatments.

### RNA isolation and real-time PCR

To quantitate gene expression and IBV replication from IBV-infected CEK cells and chicken embryos, primers and probes specific for chMDA5 [23], chIFN-β [24], chIFN-λ, chMx [25] and IBV 5′-UTR (Table 1) were used for real-time PCR as previously described [26]. Briefly, RNA was extracted using an RNA extraction kit (MiniBEST Universal RNA Extraction Kit, Takara, China) according to the manufacturer’s instructions. A total of 1 μg of RNA was then reverse transcribed to cDNA using a reverse transcription kit (HiScript Q RT SuperMix for qPCR, Vazyme, China) according to the manufacturer’s instructions, after which the transcribed products were diluted and stored at −20 °C. Gene expression was quantitated using a LightCycler 2.0 System (Roche Diagnostics Ltd., Switzerland). The relative expression ratios of the target genes chMDA5, chIFN-β, chIFN-λ and chMx were calculated using the △△Ct method. To assess IBV replication *in ovo* and in vitro, real-time PCR was performed by absolute quantitation PCR [26].

**Table 1:** SiRNA for silencing as well as primers for plasmid construction and real-time PCR used in this study

Purpose	Name	Sequence(5’ to3’)	Accession no.	References
Cloning of chMDA5	chMDA5-F1	AAAGATATCTATGTCGGAGGAGTGCCGA (*Eco*RV)	GU570144.1	
	chMDA5-R1	AATGGATCCCTTCTTTTGTCATC		
Cloning of chMDA5	chMDA5-F2	ACAAAAGAAGGGATCCATTTAGAG (overlap sequence)		
	chMDA5-R2	CTAGTCTAGATTAATCTTCATCACTTGAAGGACAA (*Xba*I)		
Cloning of chTLR3	chTLR3-F	ATAAGAATGCGGCCGCTAAACTAATGGGATGCTCTATTCCTTGCT (*Not*I)	NM_001011691	
	chTLR3-R	AAAGATATCAATCAGCGCACTTTACTATTAGATTTAAG (*Eco*RV)		
Cloning of dgRIG- I	dgRIG-I-F	GGAATTCC ATGACGGCGGAGGAAAAG (*Eco*RI)	JF804977.1	
	dgRIG-I-R	GAGGATCCTCAAATGGTGGGTACAAGTTGGAC (*Bam*HI)		
Silencing	SiMDA5	GAACGUGAAGAUGUAAAUATT		[22]
Silencing	SiTLR3	GCAGAUUGUAGUCACCUAATT		[22]
Silencing	SiMAVS	UACAGGAGGCUUCAAGGAGGUGUCA		[22]
Silencing	siRNA control	AUUACGGGCCAGUAAUCUAT		
Real-time PCR	chIFN-β F	CAGCTCTCACCACCACCTTCTC		[24]
	chIFN-β R	GGAGGTGGAGCCGTATTCTG		
Real-time PCR	chβ-actin F	CAACACAGTGCTGTCTGGTGGTA		[27]
	chβ-actin R	ATCGTACTCCTGCTTGCTGATCC		
Real-time PCR	chIFN-λ F	TGAGCTGGACCTCACCATCA	NM_001128496.1	
Real-time PCR	chIFN-λ R	GGGCTGTTGGCACGTCTCT		
Real-time PCR	chMda5 F	TGGAGCTGGGCATCTTTCAG		[23]
	chMda5 R	GTTCCCACGACTCTCAATAACAGT		
Real-time PCR	chMx F	TTGTCTGGTGTTGCTCTTCCT		[25]
	chMx R	GCTGTATTTCTGTGTTGCGGTA		
Real-time PCR	IBV-GL533	GCCATGTTGTCACTGTCTATTG		[26]
	IBV-GU391	GCTTTTGAGCCTAGCGTT		
	IBV-Probe	FAM-CACCACCAGAACCTGTCACCTC-BHQ		

The underlined nucleotides are restriction enzyme sequences. Restriction enzymes are indicated in parentheses

### Selection of appropriate reference genes

Eight housekeeping were screened to identify the most stably expressed reference genes in different chicken embryo tissues: β-actin (ACTB) [27], testis-specific alpha-tubulin mRNA (TUBAT) [28], Mitochondrial ribosomal protein S30 (MRPS30) [29], Eukaryotic translation elongation factor 1 alpha 2 (EFF1) [29], Guanine nucleotide binding protein (G protein), ribosomal protein L32 [30], β-glucuronidase (GUSB) [31], glyceraldehyde-3-phosphate dehydrogenase (GAPDH) [31] and Ribosomal protein L5 (RPL5) [29]. To calculate the stability of reference genes, three different analysis methods (geNorm, NormFinder and BestKeeper) were used [30–32]. (Additional file 1: Methods and Table S1).

### Western blot

CEK cells were infected with IBV, and at different time points post-infection, the cells were lysed with RIPA lysis buffer (Beyotime Institute of Biotechnology, China). The cell lysates were analyzed for N proteins by Western blot with anti-N antibody (1:1000) (Prepared by our laboratory). Actin was detected using a β-actin antibody (1:5000, Sigma) as a protein loading control. An anti-MAVS antibody (1:1000, Cell Signaling Technology) was used to detect the MAVS protein.

### Animal experiment

Eleven-day-old SPF chicken embryos were inoculated with IBV at 10^3^ EID_50_ via allantoic cavity. Three embryos inoculated with PBS served as a negative control. Three embryos from each group were killed at 72 h post-inoculation to determine chMDA5, chIFN-β, chIFN-λ and antiviral protein chMx transcription levels in the trachea, lung, liver, kidney, muscle and intestine of embryos, with all tissues being immediately processed for RNA extraction.

### Cell experiment

**Fig. 1:**
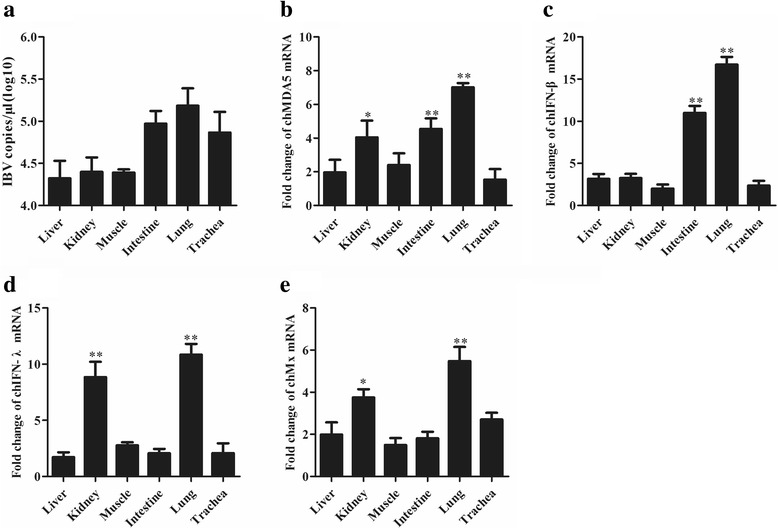
IBV induces chMDA5, chIFN-β, chIFN-λ and chMx expression in chicken embryos. In this experiment three embryos were inoculated with IBV, and three embryos were inoculated with PBS served as negative control; then, the trachea, intestine, kidney, lung, liver, and muscle tissues were collected from the embryos 72 h post-infection. **a** The IBV genome loads were quantified by RT-qPCR. **b** chMDA5, (**c**) chIFN-β, (**d**) chIFN-λ and (**e**) chMx were calculated as fold change of the infected group relative to the uninfected group and normalized against β-actin. Data are shown as the mean ± SD (*n* = 3, 3 embryos) (* *P* ≤ 0.05; ** *P* ≤ 0.01). The representatives of three independent experiments showed similar results. Values represent the average of the results from three independent experiments with standard error bars

**Fig. 2:**
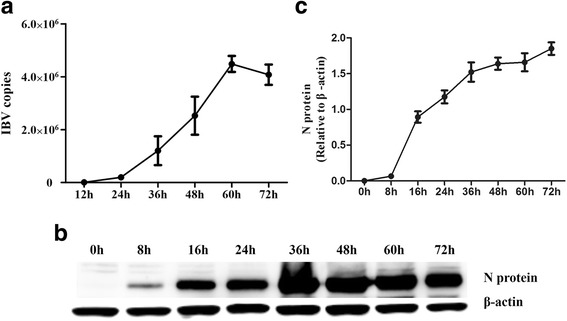
IBV replication has a time-dependent activity in CEK cells. CEK cells were infected with IBV at an MOI of 1. At the indicated times post-infection, (**a**) viral RNA was quantified by RT-qPCR. Data are presented as the mean ± SD (* *P* ≤ 0.05; ** *P* ≤ 0.01); (**b**) The cellular IBV N proteins were quantified by Western blot. **c** The graph indicating the fold change of the N proteins. The fold change of N proteins is expressed as densitometric units (Image-Pro-plus 6.0) of bands normalized to the β-actin, results from three independent experiments

CEK cells were infected with JS/2010/12 and harvested at 72 h post-inoculation. Viral stocks were prepared by freezing/thawing cells three times; the initial JS/2010/12 stock inoculated CEK cells for 5 passages. Then, DF1 cells were infected with this viral stock, and RNA was extracted at different time points. The viral genome copy number was quantified by qPCR. Although no CPE was observed, an increase in the viral genome copy number can be seen over time. CEK cells in 24-well plates were infected with IBV at an MOI of 1 and were incubated at 37 °C for 1 h. Cells that were inoculated with PBS served as a negative control. At different time points post-infection (Figs. 1 and 2), supernatants from three different wells for each group were harvested for RNA extraction to determine the level of virus replication (Fig. 1) and chMDA5, chIFN-β, chIFN-λ and chMx expression. After reaching 90% confluence, CEK cells in 6-well plates were infected with IBV at an MOI of 1. Then, cells were harvested at different time points and subsequently lysed with lysis buffer. The level of IBV replication was assessed via a Western blot assay.

### Statistical analysis

All statistical analysis were performed in GraphPad Prism 5.0. To identify significant differences between different groups, mean comparisons were performed using one-way ANOVA or student t-tests. Results were considered significant at p< 0.05.

## Results

### IBV induces IFN response in different tissues of chicken embryos

**Fig. 3:**
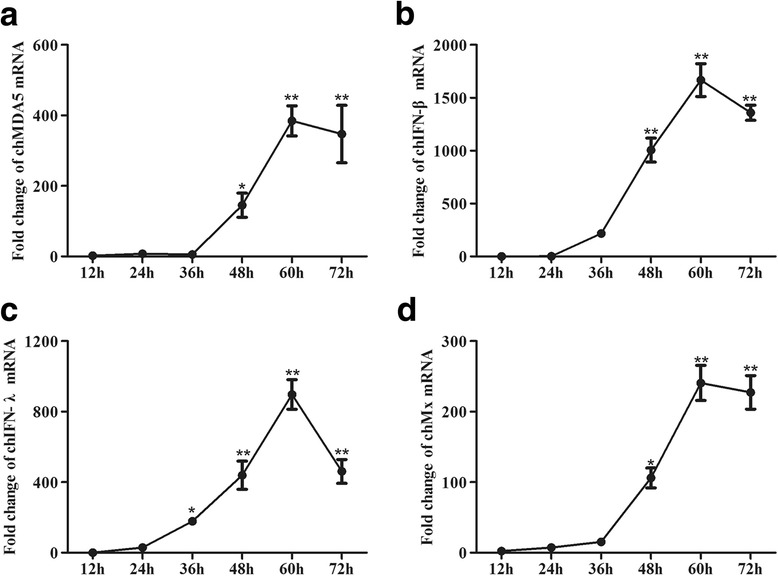
IBV induces high expression of chMDA5, chIFN-β, chIFN-λ and chMx in CEK cells. CEK cells were infected with IBV at an MOI of 1. At the indicated times post-infection, cells were harvested for RNA isolation, and virus-induced expression of chMDA5 (**a**), chIFN-β (**b**), chIFN-λ (**c**) and chMx (**d**) was determined by RT-qPCR. All gene expression was calculated as the fold change relative to mock cells uninfected in parallel and normalized against a housekeeping gene (β-actin). Data are presented as the mean ± SD (*n* = 3, 3 wells on same plate) (* *P* ≤ 0.05; ** *P* ≤ 0.01)

IBV could replicate sufficiently in 9–11 day old chicken embryos, to evaluate the relationship between antiviral response and viral replication in different embryo tissues, chicken embryos were inoculated with IBV, then the trachea, intestine, kidney, lung, liver, and muscle of the embryos were collected 72 h post-infection. The replication ability of IBV was determined by absolute quantification real-time PCR. IBV can be detected in all tissues, and the viral genome load was higher in the lung and trachea compared with other tissues (Fig. 3a). Eight housekeeping genes were selected for screening stably expressed reference genes in different chicken embryo tissues to be used in comparison to the determined cytokine expression studies (Additional file 2: Figure S1). The transcription of the antiviral cytokines chMDA5, chIFN-β, chIFN-λ and chMx in different tissues was normalized using three different reference genes (Additional file 2: Figure S1). We found that variability in expression for each gene was similar when normalized to different stable reference genes (Additional file 3: Figure S2). Therefore, β-actin was selected as an internal reference gene in this study. The data showed that chMDA5 (Fig. 3b) was expressed in all tissues, and stronger expression was observed in the intestine and lung (*P* ≤ 0.01) and kidney (*P* ≤ 0.05). IBV induced higher chIFN-β (Fig. 3c), chIFN-λ (Fig. 3d) and chMx (Fig. 3e) transcription in the lung; chMx and chIFN-λ transcription in kidneys than the negative control group (*P* ≤ 0.01).

### Accumulation of a large amount of dsRNA in IBV-infected CEK cells results in a strong IFN response

To investigate the replication ability of IBV in vitro, CEK cells were infected with IBV at an MOI of 1. The replication of IBV was quantified by RT-qPCR to detect the IBV genome load in cell culture supernatants, and N protein was detected by a Western blot assay. We observed a significant increase in the IBV genome load in cell culture supernatants, with the highest level occurring at 60 h post-infection for the IBV-infected cells compared to the other time points (Fig. 1a). The IBV N protein expression data are illustrated in Fig. 1b and c. The highest expression level of N protein occurred at 72 h post-infection.

To monitor the kinetics of the chMDA5 and IFN response in relation to IBV replication in vitro, the transcription of chMDA5, chIFN-β, chIFN-λ and chMx was quantified in IBV-infected CEK cells (Fig. 2a–d). The expression of chMDA5, chIFN-β, chIFN-λ and chMx peaked at 60 h post-infection. The chIFN-β transcription level was significantly down regulated at 12 h post-infection and upregulated from 24 h to 60 h post-infection. IBV-infected CEK cells exhibited a significant induction of innate immunity gene transcription (chMDA5, chIFN-β, chIFN-λ and chMx) compared with the negative control group at 48 h, 60 h and 72 h (*P* ≤ 0.05) post-infection, consistent with IBV replication.

### Dose-dependent antiviral cytokine potency of IBV

**Fig. 4:**
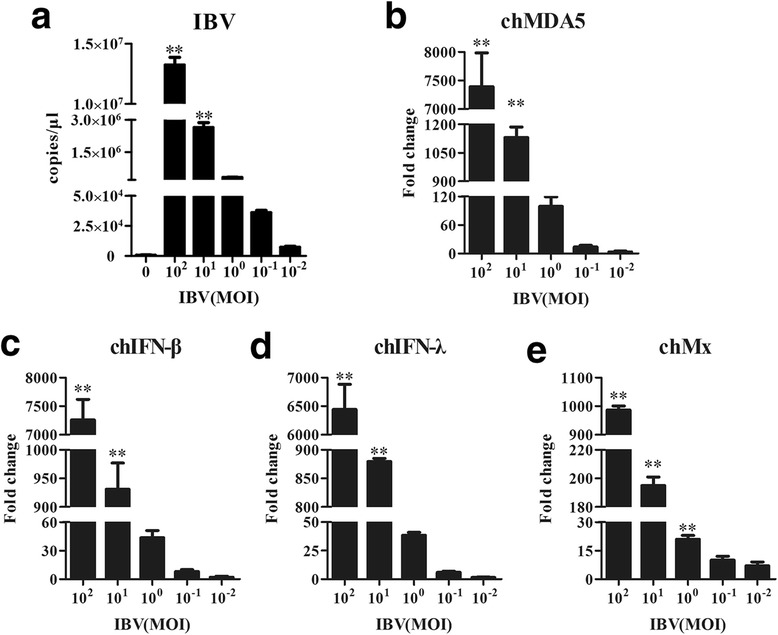
Dose-dependent antiviral cytokine potency of IBV. CEK cells were inoculated with 10^−2^, 10^−1^, 10^0^, 10^1^ and 10^2^ MOI of IBV, then the cells were harvested at 36 h post-treatment for RNA extraction. The IBV genome load (**a**) was quantified by RT-qPCR. The mRNA levels of chMDA5 (**b**), chIFN-β (**c**), chIFN-λ (**d**) and chMx (**e**) were evaluated by RT-qPCR. All gene expression was calculated as the fold change relative to uninfected control cells and normalized against a housekeeping gene (β-actin). Data are presented as the mean ± SD (n = 3, 3 wells on same plate) (* *P* ≤ 0.05; ** *P* ≤ 0.01)

To determine the relationship between the antiviral immune response and virus titer, CEK cells were infected with IBV at 10^−2^, 10^−1^, 10^0^, 10^1^ and 10^2^ MOI for 36 h. As shown in Fig. 4, IBV induced chMDA5 (Fig. 4b), chIFN-β (Fig. 4c), chIFN-λ (Fig. 4d) and chMx (Fig. 4e) transcription in CEK cells at different infectious doses. We also found that IBV induced chMDA5, chIFN-β, chIFN-λ and chMx transcription in CEK cells to a greater extent than in mock-infected cells at infectious doses of 10^1^ and 10^2^ MOI (*P* ≤ 0.01). Overall, IBV significantly induced the activation of chMDA5, chIFN-β, chIFN-λ and chMx in a dose-dependent manner.

### ChMDA5 and dgRIG-I enhance the transcription of chIFN-β induced by IBV in CEK cells

**Fig. 5:**
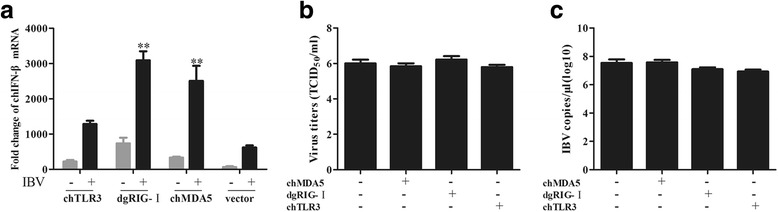
DgRIG- I and chMDA5 overexpression enhances IBV-induced IFN-β transcription. **a** DF1 cells were transfected with the indicated plasmid for 24 h and then infected with IBV for 24 h. The extracted RNA was used to measure the expression of IFN-β. The expression of IFN-β in the test group was compared to the mock control group that was transfected with the control vector and infected with IBV. The growth properties of IBV in chTLR3-, chMDA5- and dgRIG-I-overexpressed cell supernatants titrated onto CEK cells are expressed as TCID_50_/ml (**b**) or by RT-qPCR to determine IBV genome load (**c**). Experiments were performed in triplicate, and data are representative of three independent experiments (* P ≤ 0.05; ** P ≤ 0.01)

The PRRs RIG-I and MDA5 are critical regulators of the host antiviral response and share a similar homology in their overall primary structure. This similarity prompted us to investigate whether dgRIG-I has the same role as chMDA5 in the IBV-induced IFN response in chicken cells. The expression of chMDA5 in DF1 cells enhanced chIFN-β transcription after the cells were infected with IBV compared with the control group transfected with vector and primed by IBV infection (*P* ≤ 0.05) (Fig. 5a). We also found that infection with IBV induced higher chIFN-β transcription in dgRIG-I-expressing cells to a greater extent than the control group (*P* ≤ 0.01), and dgRIG-I induced chIFN-β expression after IBV infection to a greater extent than chMDA5. Expression of chTLR3 did not enhance chIFN-β transcription after being infected with IBV. These results appear to indicate that IBV stimulates chIFN-β transcription via MDA5. Similarly, the IBV-induced chIFN-β transcription was further increased by the overexpression of dgRIG-I. These data show that chMDA5 and dgRIG-I act as positive regulators of the IBV-induced chIFN-β signaling pathway (Fig. 5a).

To investigate whether chMDA5 and dgRIG-I can influence the replication of IBV, DF1 cells were transfected with chMDA5 or dgRIG-I, then were infected with IBV at an MOI of 1. The supernatants were harvested and titrated onto CEK cells. The test results indicated that neither the expression of chMDA5 nor dgRIG-I in CEK cells affected viral replication (Fig. 5b and c).

### IBV cleaves MAVS at an early stage of infection

**Fig. 6:**
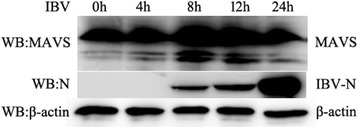
IBV cleaves MAVS at an early stage of infection. CEK cells were infected with IBV at an MOI of 1. At the indicated times post-infection, cells were lysed and the expression of MAVS proteins were analyzed by Western blot using an anti-MAVS antibody (top panel). The IBV-encoded N protein was detected with an anti-N antibody (second panel). β-actin was detected as a loading control (bottom panel)

RIG-I and MDA5 interact with MAVS via CARD-CARD domains at their N-terminal, which is essential for the activation of the NF-κB and IRF3/7 downstream signaling pathway. MAVS plays a critical role in antiviral activity. To determine whether chMAVS is involved in the antiviral immune response to IBV infection, the expression level of chMAVS in IBV-infected CEK cells was investigated. We found that the cleavage of MAVS was induced by IBV at 8 h and 12 h post-infection but was not cleaved after 24 h (Fig. 6). We inferred that at the early stage of IBV infection, IBV-induced cleavage of MAVS allows IBV to evade the MDA5-mediated innate immunity signaling pathway.

### Knockdown of chMDA5 and chMAVS influences IBV-induced IFN-β transcription

**Fig. 7:**
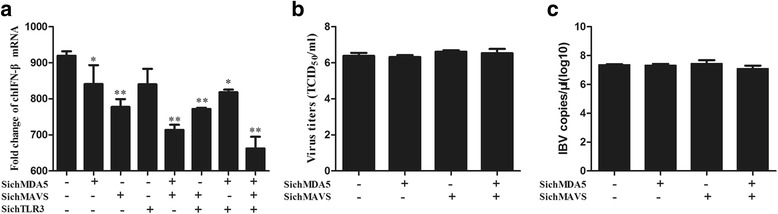
chMDA5 and chMAVS knockdown influenced IBV-induced IFN-β transcription. **a** DF1 cells were transfected with siRNA specific for chMDA5, chTLR3 or chMAVS alone or in combination for 24 h, after which the cells were infected with IBV for 24 h. The expression levels in the silenced groups were compared to the control siRNA-treated cells. The growth properties of IBV in chMDA5- and chMAVS-silenced cell supernatants titrated onto CEK cells are presented as TCID_50_/ml (**b**) or by RT-qPCR to determine IBV genome load (**c**). Data are represented as the mean ± SD from three independent experiments (* P ≤ 0.05; ** P ≤ 0.01)

A gene silencing technique was also used to identify whether chMDA5, chTLR3 and chMAVS influence the antiviral immune response mediated by IBV. First, to assess the effect of chMDA5, chTLR3 and chMAVS on IBV-induced IFN-β transcription, the expression of chMDA5, chTLR3 and chMAVS was knocked down. Compared with the control group, silencing of chMAVS or chMDA5 mRNA in DF1 cells significantly reduced IBV-induced IFN-β transcription. The simultaneous silencing of chMDA5, chTLR3 and chMAVS reduced the transcription of IFN-β significantly (Fig. 7a). These results suggest that both chMDA5 and chMAVS are involved in the activation of IFN-β in DF1 cells following IBV infection.

To further understand the role of chMDA5 and chMAVS during IBV infection, chMDA5 and chMAVS were knocked down in CEK cells, then the cells were infected with IBV at an MOI of 1. IBV in supernatants was titrated onto CEK cells at 48 h post-infection while the IBV genome loads were quantitated by real-time PCR. The results indicated that the observed viral replication in chMDA5 and chMAVS knockdown cells was the same as in the control group (Fig. 7b and c). Overall, inhibition of chMDA5 and chMAVS expression has no effect on the replication of IBV.

## Discussion

*In ovo* and in vitro study, we show that infection with IBV leads to a considerable activation of the type I IFN and antiviral immune response [33]. Among birds, the RIG-I and MDA5 genes are present in ducks, geese and pigeons, but only MDA5 gene can be identified in chickens [34]. RIG-I and MDA5 share a similar homology in their overall primary structure and induce the downstream signaling pathway involving MAVS [7, 11, 12]. In this study, to investigate the effect of dgRIG-I in the chicken immune system response to IBV infection, we identified and cloned dgRIG-I. We found that dgRIG-I plays a similar role to chMDA5 in upregulating the transcription of chIFN-β in response to IBV infection. It has been previously demonstrated that IBV can delay the transcription of chIFN-β in the early stages of infection [35]. In this study, we found that through the cleavage of the chMAVS protein, IBV can downregulate chIFN-β transcription, and the knockdown of chMAVS had a dramatic effect on chIFN-β activation, indicating a more predominant role of MAVS in the innate immune response against IBV infection.

Tissue distribution is an important characteristic of MDA5 function, as it influences the capacity of MDA5 to capture different viruses as they enter and proliferate in different tissues [22, 23]. Understanding the distribution patterns of MDA5 will enable us to explain the relationship between the immune system and viral infection, and help to identify the relationship between IBV and the host. Previous reports found that expression of the chMDA5 gene could be detected in all tissues examined [22, 23, 34]. Wenxin Zhang demonstrated that IBV failed to increase the MDA5 promoter activity and the expression of endogenous MDA5, which may be explained by the differences in virulence and adaptability of the IBV strain [36]. The expression of MDA5 was significantly upregulated in chicken intestine and lung after being infected with IBV, which in turn upregulated the expression of IFN-β and IFN-λ. Based on this distribution, chMDA5 can respond to invading pathogens as early as possible.

MDA5 and RIG-I are the two major PRRs for detecting RNA viruses. They can both detect RNA viruses and activate a signaling pathway that leads to the production of type I interferon and the initiation of antiviral activities [17, 37]. Considerable attention has recently been given to the role of chMDA5 in IBV infections in chickens, which appear to lack RIG-I [34]. Our results confirm the findings that chMDA5 is the receptor that mediates the antiviral response to IBV infection. Upregulation of chIFN-β following IBV infection in chMDA5-overexpression cells suggests that chMDA5 interacts with the proteins induced by IBV infection [25]. The silencing of chMDA5 expression resulted in a reduction of IBV-induced chIFN-β transcription and highlights the role that chMDA5 may play in antiviral defense. The absence of RIG-I in chickens may lead to insufficient antiviral responses to IBV infections, resulting in only chickens being susceptible to IBV infections, whereas ducks and geese, which possess RIG-I, are not infected with IBV [38]. chIFN-β was upregulated in response to IBV infection in dgRIG-I-overexpressed chicken cells, which is similar to observations made in mammalian models [23, 38]. Our study implies that dgRIG-I plays a similar role as chMDA5 in IBV-infected chicken cells, inducing the interferon response.

Although chMDA5 was found to be capable of sensing IBV, leading to the induction of chIFN-β expression in chicken cells, the overexpression of chIFN-β induced by chMDA5 and dgRIG-I did not affect the viral replication in our experiment. Silencing of chMDA5 also had little impact on IBV replication, and a subtle role for chMDA5 may be masked by incomplete silencing of chMDA5, resulting in trace levels of IFN activity. Nevertheless, our data gave a similar result as mammalian models showing that MDA5 is not critical to combat the influenza virus [23]. It is known that type I IFNs are induced by the infection of host cells with viruses and that secreted type I IFNs cause cells to express various antiviral proteins, such as myxovirus-resistance protein (Mx) GTPase, ribonuclease L (RNase L), RNA-dependent protein kinase (PKR), oligoadenylate synthetase (OAS), and interferon stimulated gene (ISG) by autocrine and paracrine mechanisms [27]. Previous studies have suggested that IBV replication was reduced by 50%, as measured by syncytia formation, after a treatment with 100 U/ml of IFN [39, 40]. This implies that even in cells responding to type I IFN, the autocrine and paracrine effects of IFNs from virus-infected cells may not be sufficient to suppress viral replication. IBV delays the IFN response at an early stage of infection in chicken cells, since it needs time to infect neighboring cells before the establishment of an antiviral state induced by IFN [25], which is secreted paracrinely by chMDA5 and dgRIG-I mediated induction in IBV-infected cells. The data in Fig. 4 also support this conclusion, which suggests that the expression level of IFN in the group infected with a higher dose of IBV was higher than in the group that received a lower dose.

Infectious diseases are a manifestation of constant battles between the host and pathogenic microbes. This host-pathogen antagonism is demonstrated by the interaction between viruses and MAVS, a critical molecule that is downstream of MDA5 and RIG-I [11, 41]. For example, MAVS can orchestrate a strong immune response against hepatitis C virus (HCV), but HCV counterattacks by cleaving MAVS, thus crippling the immune response [42, 43]. MAVS activity was proposed to be linked to both peroxisomes and mitochondrial location in the assembly of a macromolecular signaling complex [13]. When MAVS was cleaved and released to the outside of mitochondria, such as the endoplasmic reticulum or cytoplasm, it failed to signal [13, 44]. IBV can efficiently cleave MAVS in the early stages of infection, leading to the blockage of IFN expression. Furthermore, chIFN-β expression, induced by IBV infection, was effectively inhibited by blocking the expression of MAVS using siRNA. More significant results were observed when MAVS was silenced together with siMDA5. In conclusion, we showed that chMAVS acts as a key modulator of antiviral signaling by regulating chMDA5-mediated signaling in IBV-infected cells. Future studies should focus on more detailed aspects of host-pathogen interactions that involve chMAVS to gain control of the host immune system.

## Conclusions

Taken together, our study represents a comprehensive analysis of the host antiviral immune response against IBV infection. We show that IBV induces activation of the IFN response in CEK cells and chicken embryos. We also found that chMAVS acts as a key modulator in antiviral signaling by positively regulating chMDA5-mediated signaling. dgRIG-I and chMDA5 have similar roles in the IBV-induced IFN-β signaling pathway, the absence of RIG-I may increase the susceptibility of chickens to IBV infection. The exact contributions of these PRRs and MAVS are worth exploring in future studies.

## Additional files

Additional file 1: Supplementary information, selection of appropriate reference genes. (DOCX 36 kb)

Additional file 2: Figure S1. Transcriptional stability of eight candidate reference genes in different chicken embryo tissues. Three eleven-day-old SPF chicken embryos were inoculated with PBS. Then, the kidney (A and B), liver (C and D), muscle (E and F), intestine (G and H), lung (I and J) and trachea (K and L) tissues were collected from the embryos 72 h post-inoculation, and RNA was extracted. Candidate reference gene mRNA was amplified by real-time PCR. The transcriptional stability of the candidate reference genes was measured using geNorm and NormFinder software. (A), (C), (E), (G), (I) and (K) are the geNorm analysis results. Average expression stability M of all eight reference genes. The most stably expressed genes have lower M values. (B), (D), (F), (H), (J) and (L) are the NormFinder analysis results. The lower stability value indicates a gene that is more stable. (TIFF 730 kb)

Additional file 3: Figure S2. IBV induces chMDA5, chIFN-β, chIFN-λ and chMx expression in chicken embryos. In this experiment three embryos were inoculated with IBV, and three embryos were inoculated with PBS, which served as negative controls. Then, the trachea, intestine, kidney, lung, liver, and muscle tissues were collected from the embryos 72 h post-infection. (A) chMDA5, (B) chIFN-β, (C) chIFN-λ and (D) chMx were calculated as fold change of the infected group relative to the uninfected group and normalized against three different reference genes. In the kidney, liver and muscle tissues chMDA5, chIFN-β, chIFN-λ and chMx transcription was normalized to ACTB (1), EFF1 (2) and RPL5 (3) respectively. In the intestine chMDA5, chIFN-β, chIFN-λ and chMx transcription was normalized to ACTB (1), GAPDH (2) and RPL32 (3) respectively. In the lung chMDA5, chIFN-β, chIFN-λ and chMx transcription was normalized to ACTB (1), TUBAT (2) and RPL5 (3) respectively. In the trachea chMDA5, chIFN-β, chIFN-λ and chMx transcription was normalized to ACTB (1), EFF1 (2) and RPL32 (3) respectively. Data are shown as the mean ± SD. (*n*=3) (* *P* ≤ 0.05; ** *P* ≤ 0.01). (TIFF 1330 kb)

Additional file 4: The datasets analysed during the current study. (XLSX 20 kb)

## Abbreviations

ACTB: β-actin
AIV: Avian influenza virus
CARD: Caspase activation and recruitment domains
CEK: Chicken embryonic kidney
CPE: Cytopathic effect
EFF1: Eukaryotic translation elongation factor 1 alpha
EMCV: Encephalomyocarditis virus
GAPDH: Glyceraldehyde-3-phosphate dehydrogenase
GUSB: β-glucuronidase
HCV: Hepatitis C virus
IBV: Infectious bronchitis virus
IFN: Interferon
IFN-λ: Interferon-lambda
IRF3/IRF7: Interferon regulatory factor-3/7
ISG: Interferon stimulated gene
LGP2: Laboratory of genetics and physiology 2
MAVS: Mitochondrial antiviral signaling gene, also called IPS-1/VISA/CARDIF
MDA5: Melanoma differentiation associated gene 5
MOI: Multiplicity of infection
MRPS30: Mitochondrial ribosomal protein S30
MV: Measles virus
Mx: Myxovirus-resistance protein
NLRs: Nucleotide-binding oligomerization domain (NOD)-like receptors
OAS: Oligoadenylate synthetase
PAMPs: Pathogen-Associated molecular patterns
PKR: RNA-dependent protein kinase
PRRs: Pattern recognition receptors
RIG-I: Retinoic acid-inducible gene-I
RLRs: Retinoic acid-inducible gene I -like receptors
RNase L: Ribonuclease L
RPL32: Guanine nucleotide binding protein (G protein), ribosomal protein L32
RPL5: Ribosomal protein L5
siRNA: Small interfering RNA
SPF: Specific pathogen free
TCID50: 50% tissue culture infective dose
TUBAT: Testis-specific alpha-tubulin mRNA
VSV: Vesicular stomatitis virus
