# Supplementary material for: Avian infectious bronchitis virus disrupts the melanoma differentiation associated gene 5 (MDA5) signaling pathway by cleavage of the adaptor protein MAVS
Source: BMC Vet Res. 2017 Nov 13;13:332. doi: 10.1186/s12917-017-1253-7 (PMC5683607; doi:10.1186/s12917-017-1253-7)
Supplement: Supplementary file 1 — Supplementary information, selection of appropriate reference genes. (DOCX 36 kb) [file 12917_2017_1253_MOESM1_ESM.docx]

**Supplementary information**

**Methods**

**Selection of appropriate reference genes**

Three eleven-day-old SPF chicken embryos were inoculated with PBS. Then, the kidney, liver, muscle, intestine, lung and trachea tissues were collected from the embryos 72 h post-inoculation, and RNA was extracted. Eight genes used as reference genes to identify the most stably expressed reference genes in different tissues were selected: β-actin (ACTB) [[1](#_ENREF_1)], testis-specific alpha-tubulin mRNA (TUBAT) [[2](#_ENREF_2)], Mitochondrial ribosomal protein S30 (MRPS30) [[3](#_ENREF_3)], Eukaryotic translation elongation factor 1 alpha 2 (EFF1) [[3](#_ENREF_3)], Guanine nucleotide binding protein (G protein), ribosomal protein L32 (RPL32) [[4](#_ENREF_4)], β-glucuronidase (GUSB) [[5](#_ENREF_5)], glyceraldehyde-3-phosphate dehydrogenase (GAPDH) [[5](#_ENREF_5)] and Ribosomal protein L5 (RPL5) [[3](#_ENREF_3)]. Quantitative real-time PCR reactions were performed using the SYBRGreen quantification method with LightCycler FastStart DNA Master PLUS SYBR Green I (Roche Diagnostics Ltd, Switzerland). All reactions were performed on a [LightCycler^®^ 2.0 Instrument](https://shop.roche.com/shop/en/global/products/lightcycler14301-20-instrument) (Roche Diagnostics Ltd, Switzerland). The average Cq values of reference genes were used to set the input files according to the software programs. To calculate the stability of reference genes, three different analysis methods (geNorm, NormFinder and BestKeeper [[4-6](#_ENREF_4)]) were used. All data were analyzed according to the instructions for the software tested. The relative stability of eight candidate genes was ranked by geNorm M value and NormFinder stability value with the more stable genes having the lowest value. BestKeeper uses Cq as input to calculate descriptive statistics, and standard deviation values for stable references need less than one.

1. Boo KH, Yang JS: **Intrinsic cellular defenses against virus infection by antiviral type I interferon**. *Yonsei medical journal* 2010, **51**(1):9-17.

2. Nascimento CS, Barbosa LT, Brito C, Fernandes RP, Mann RS, Pinto AP, Oliveira HC, Dodson MV, Guimaraes SE, Duarte MS: **Identification of Suitable Reference Genes for Real Time Quantitative Polymerase Chain Reaction Assays on Pectoralis major Muscle in Chicken (Gallus gallus )**. *PloS one* 2015, **10**(5):e0127935.

3. Cedraz de Oliveira H, Pinto Garcia AAJ, Gonzaga Gromboni JG, Vasconcelos Farias Filho R, Souza do Nascimento C, Arias Wenceslau A: **Influence of heat stress, sex and genetic groups on reference genes stability in muscle tissue of chicken**. *PloS one* 2017, **12**(5):e0176402.

4. Bages S, Estany J, Tor M, Pena RN: **Investigating reference genes for quantitative real-time PCR analysis across four chicken tissues**. *Gene* 2015, **561**(1):82-87.

5. Borowska D, Rothwell L, Bailey RA, Watson K, Kaiser P: **Identification of stable reference genes for quantitative PCR in cells derived from chicken lymphoid organs**. *Veterinary immunology and immunopathology* 2016, **170**:20-24.

6. Batra A, Maier HJ, Fife MS: **Selection of reference genes for gene expression analysis by real-time qPCR in avian cells infected with infectious bronchitis virus**. *Avian Pathol* 2017, **46**(2):173-180.

**Supplementary Fig. 1. Transcriptional stability of eight candidate reference genes in different chicken embryo tissues.** Three eleven-day-old SPF chicken embryos were inoculated with PBS. Then, the kidney (A and B), liver (C and D), muscle (E and F), intestine (G and H), lung (I and J) and trachea (K and L) tissues were collected from the embryos 72 h post-inoculation, and RNA was extracted. Candidate reference gene mRNA was amplified by real-time PCR. The transcriptional stability of the candidate reference genes was measured using geNorm and NormFinder software. (A), (C), (E), (G), (I) and (K) are the geNorm analysis results. Average expression stability M of all eight reference genes. The most stably expressed genes have lower M values. (B), (D), (F), (H), (J) and (L) are the NormFinder analysis results. The lower stability value indicates a gene that is more stable.

**Supplementary Fig. 2. IBV induces chMDA5, chIFN-β, chIFN-λ and chMx expression in chicken embryos.** In this experiment three embryos were inoculated with IBV, and three embryos were inoculated with PBS, which served as negative controls. Then, the trachea, intestine, kidney, lung, liver, and muscle tissues were collected from the embryos 72 h post-infection. (A) chMDA5, (B) chIFN-β, (C) chIFN-λ and (D) chMx were calculated as fold change of the infected group relative to the uninfected group and normalized against three different reference genes. In the kidney, liver and muscle tissues chMDA5, chIFN-β, chIFN-λ and chMx transcription was normalized to ACTB (1), EFF1 (2) and RPL5 (3) respectively. In the intestine chMDA5, chIFN-β, chIFN-λ and chMx transcription was normalized to ACTB (1), GAPDH (2) and RPL32 (3) respectively. In the lung chMDA5, chIFN-β, chIFN-λ and chMx transcription was normalized to ACTB (1), TUBAT (2) and RPL5 (3) respectively. In the trachea chMDA5, chIFN-β, chIFN-λ and chMx transcription was normalized to ACTB (1), EFF1 (2) and RPL32 (3) respectively. Data are shown as the mean ± SD. (n=3) (* P ≤0.05; ** P ≤0.01).

**Supplementary Table 1. Candidate reference genes used for the real-time PCR experiment.**

| Gene | Name | Sequence (5’ to 3’) | References |
| --- | --- | --- | --- |
| GUSB | GUSB-F | GGCAGACTGGTCCTGTTGTTG | [[5](#_ENREF_5)] |
|  | GUSB-R | GGGTCCTGAGTGATGTCATTGA |  |
| GAPDH | GAPDH-F | GAAGGCTGGGGCTCATCTG | [[5](#_ENREF_5)] |
|  | GAPDH-R | CAGTTGGTGGTGCACGATG |  |
| RPL32 | RPL-32-F | ATGGGAGCAACAAGAAGACG | [[4](#_ENREF_4)] |
|  | RPL-32-R | TTGGAAGACACGTTGTGAGC |  |
| MRPS30 | MRPS30-F | CCTGAATCCCGAGGTTAACTATT | [[3](#_ENREF_3)] |
|  | MRPS30-R | GAGGTGCGGCTTATCATCTATC |  |
| EFF1 | EFF1-F | GCCCGAAGTTCCTGAAATCT | [[3](#_ENREF_3)] |
|  | EFF1-R | AACGACCCAGAGGAGGATAA |  |
| TUBAT | TUBAT-F | CAGCTCTCAGTGGCTGAAATCA | [[2](#_ENREF_2)] |
|  | TUBAT-R | CCTTGTTGCGGGTCACACTT |  |
| RPL5 | RPL5-F | AATATAACGCCTGATGGGATGG | [[3](#_ENREF_3)] |
|  | RPL5-R | CTTGACTTCTCTCTTGGGTTTCT |  |
