# Supplementary figures and images for: Avian infectious bronchitis virus disrupts the melanoma differentiation associated gene 5 (MDA5) signaling pathway by cleavage of the adaptor protein MAVS
Source: BMC Vet Res. 2017 Nov 13;13:332. doi: 10.1186/s12917-017-1253-7 (PMC5683607; doi:10.1186/s12917-017-1253-7)

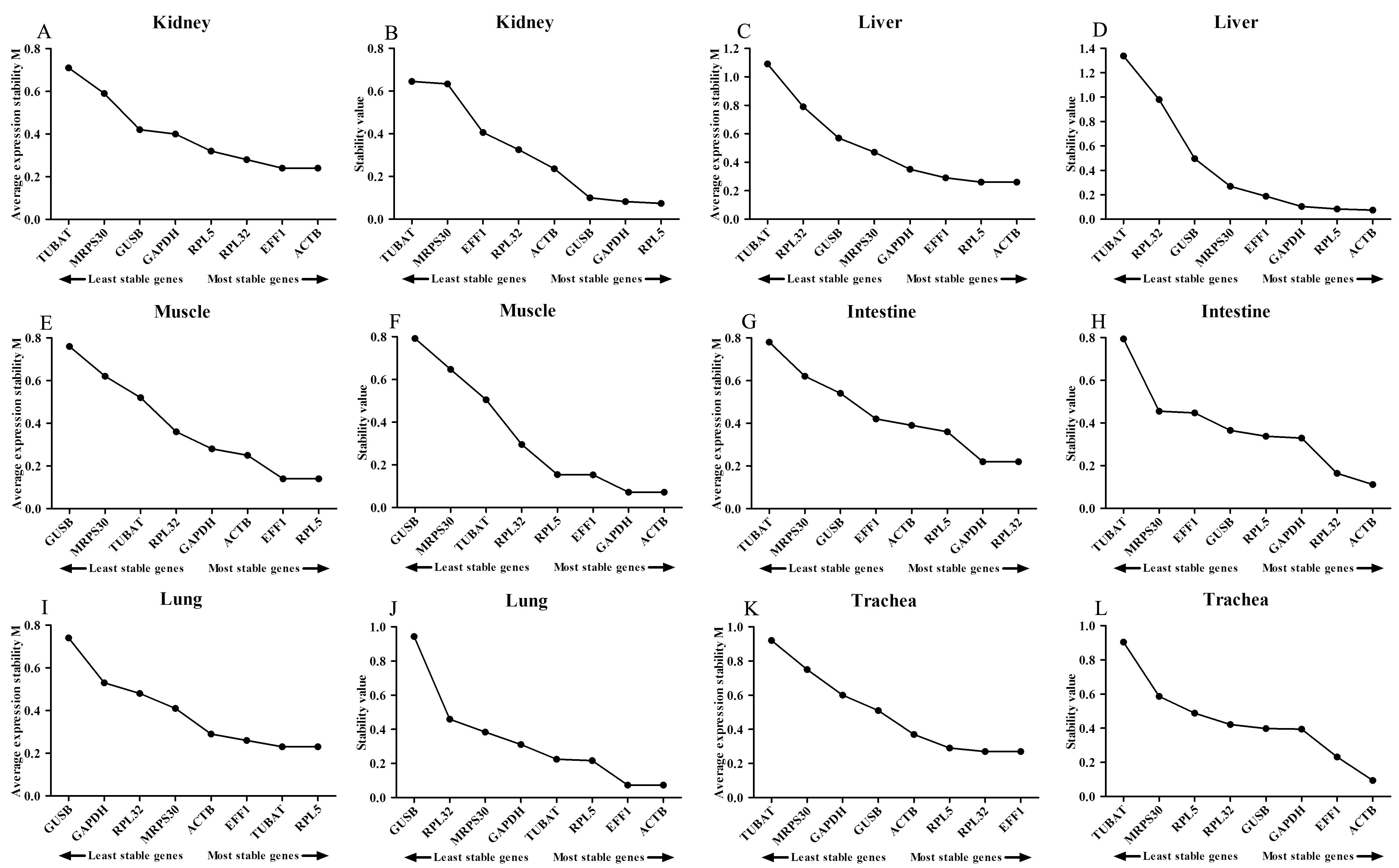

Supplement: Supplementary file 2 — Transcriptional stability of eight candidate reference genes in different chicken embryo tissues. Three eleven-day-old SPF chicken embryos were inoculated with PBS. Then, the kidney (A and B), liver (C and D), muscle (E and F), intestine (G and H), lung (I and J) and trachea (K and L) tissues were collected from the embryos 72 h post-inoculation, and RNA was extracted. Candidate reference gene mRNA was amplified by real-time PCR. The transcriptional stability of the candidate reference genes was measured using geNorm and NormFinder software. (A), (C), (E), (G), (I) and (K) are the geNorm analysis results. Average expression stability M of all eight reference genes. The most stably expressed genes have lower M values. (B), (D), (F), (H), (J) and (L) are the NormFinder analysis results. The lower stability value indicates a gene that is more stable. (TIFF 730 kb) [file 12917_2017_1253_MOESM2_ESM.tif]

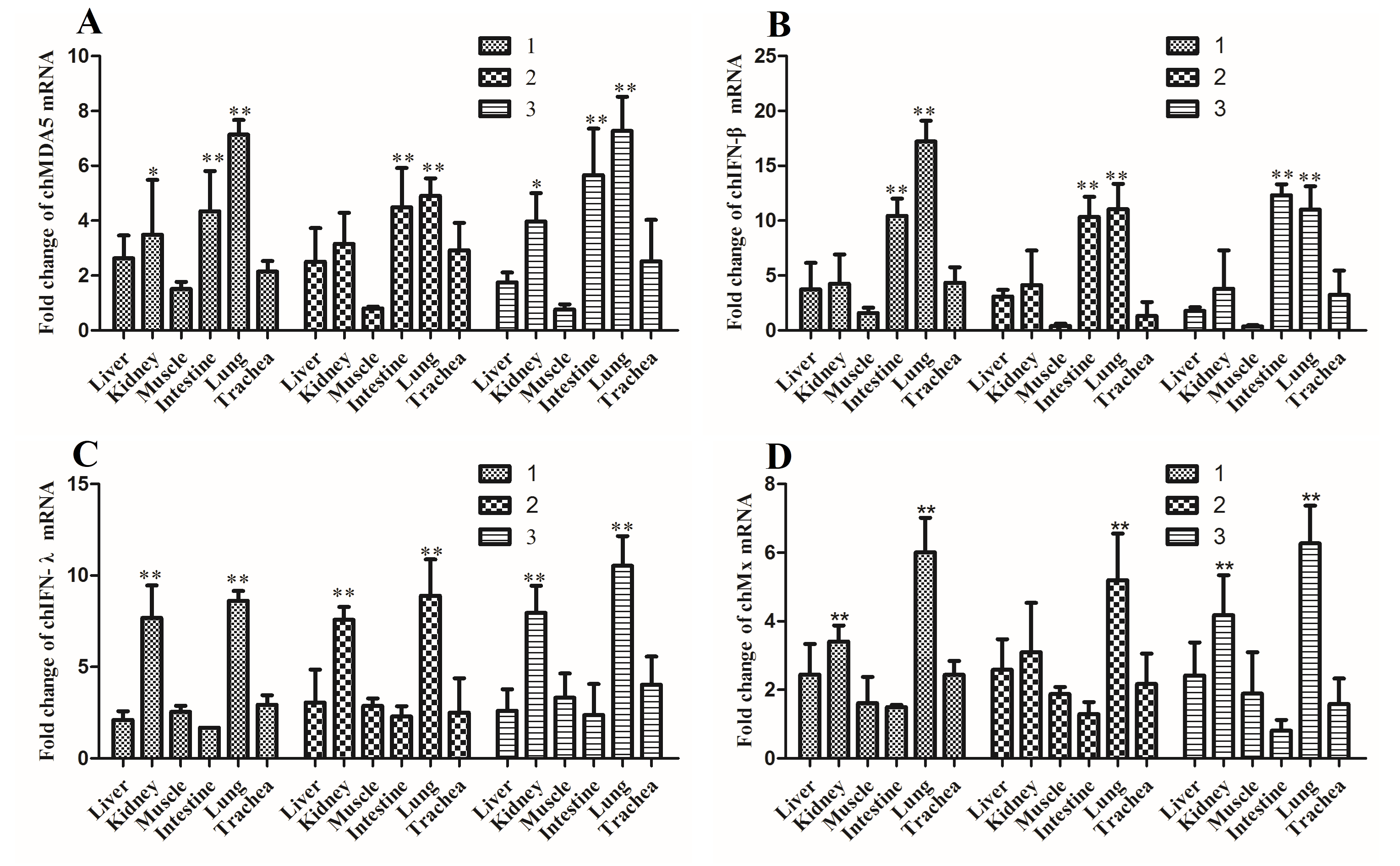

Supplement: Supplementary file 3 — IBV induces chMDA5, chIFN-β, chIFN-λ and chMx expression in chicken embryos. In this experiment three embryos were inoculated with IBV, and three embryos were inoculated with PBS, which served as negative controls. Then, the trachea, intestine, kidney, lung, liver, and muscle tissues were collected from the embryos 72 h post-infection. (A) chMDA5, (B) chIFN-β, (C) chIFN-λ and (D) chMx were calculated as fold change of the infected group relative to the uninfected group and normalized against three different reference genes. In the kidney, liver and muscle tissues chMDA5, chIFN-β, chIFN-λ and chMx transcription was normalized to ACTB (1), EFF1 (2) and RPL5 (3) respectively. In the intestine chMDA5, chIFN-β, chIFN-λ and chMx transcription was normalized to ACTB (1), GAPDH (2) and RPL32 (3) respectively. In the lung chMDA5, chIFN-β, chIFN-λ and chMx transcription was normalized to ACTB (1), TUBAT (2) and RPL5 (3) respectively. In the trachea chMDA5, chIFN-β, chIFN-λ and chMx transcription was normalized to ACTB (1), EFF1 (2) and RPL32 (3) respectively. Data are shown as the mean ± SD. (n=3) (* P ≤ 0.05; ** P ≤ 0.01). (TIFF 1330 kb) [file 12917_2017_1253_MOESM3_ESM.tif]
